# Accuracy of Mini-Implant Placement Using a Computer-Aided Designed Surgical Guide, with Information of Intraoral Scan and the Use of a Cone-Beam CT

**DOI:** 10.3390/dj10060104

**Published:** 2022-06-08

**Authors:** Georgios Vasoglou, Ioanna Stefanidaki, Konstantinos Apostolopoulos, Evmorfia Fotakidou, Michail Vasoglou

**Affiliations:** 1 Department of Orthodontics, 401 General Military Hospital, 138 Mesogeion Avenue, 11525 Athens, Greece; gio.vasoglou@gmail.com (G.V.); efident@yahoo.com (E.F.); 2 Private Orthodontic Practice, 74 Mantzagriotaki Str, Kallithea, 17676 Athens, Greece; ioanna.stefanidaki@gmail.com; 3 Orthodontics Department, School of Dental Medicine, Case Western Reserve University, 10900 Euclid Ave, Cleveland, OH 44106, USA; konapost127@gmail.com; 4 Department of Orthodontics, School of Dentistry, National and Kapodistrian University of Athens, 2 Thivon Str, Goudi, 11527 Athens, Greece

**Keywords:** mini-implants, surgical-guide, cone-beam CT, 3D-printing

## Abstract

Background: The purpose of the study was to investigate the accuracy of mini-implant placement with the use of a computer designed surgical guide derived by intraoral scanning alongside Cone-Beam Computed Tomography (CBCT) or the use of a 2D radiograph. Methods: Thirty-five mini-implants (Aarhus^®^ System: *n* = 20, Ø: 1.5 mm and AbsoAnchor^®^: *n* = 15, Ø: 1.3–1.4 mm) were placed in the maxilla and mandible of 15 orthodontic patients for anchorage purposes in cases where a CBCT was needed due to impacted teeth or for safety reasons. All were placed with the help of a computer designed surgical guide. One implant became loose and was excluded from the study. For 18 mini-implants (study group), CBCT and intraoral scanning were used for guide design, while for 16 (control group) only intraoral scanning and panoramic imaging information were used. Mini-implant position was recorded by angular and linear measurements on digital models created by combining Digital Imaging and Communications in Medicine (DICOM) and stereolithography (.stl) files. Accuracy in positioning was determined by comparing corresponding measurements for virtual and real positioned mini-implants on digital models before and after operation. The results were statistically analyzed with t-tests and the Mann-Whitney test. Results: No significant statistical differences were found for pre- and post-operational angular measurements in the study group, while significant statistical differences occurred on the same measurements for the control group (coronal angle 13.6° pre-op and 22.7° post-op, *p*-value = 0.002, axial angle 13.4° pre-op and 15.9° post-op, *p*-value = 0.034). Linear measurements pre- and post-operational for either group presented no significant statistical differences. Conclusions: A 3D designed and manufactured surgical guide with information concerning CBCT and intraoral scanning ensures accuracy on mini-implant placement while design of the guide without the use of a CBCT is less accurate, especially on inclination of the implant.

## 1. Introduction

Innovative devices and technologies to reduce morbidity, biological, and surgical times, are an intense research topic in the implant dentistry [1]. Mini implants were introduced in orthodontics as devices for enhancing anchorage almost two decades ago [2,3,4,5,6]. However, in the beginning, the importance of precise implementation was not known. It is now accepted that the following factors may compromise the desired performance of the implant: the point of insertion in the mucosa, the angle to the occlusal plane, the length, and the exact position among roots regarding tooth movement [7,8]. Inaccurate positioning can lead to root or even sinus perforation, insufficient primary stability and mini-implant failure; thus, recommended sites for insertion [9] and recommended torque placement have been proposed [10].

Various methods have been described in the literature for accurate positioning of orthodontic mini-implants: surgical guides fabricated by acrylic resin on patients’ dental casts [11,12,13], wire stents for accurate implantation [14,15] and a stainless-steel tube for mini-implant guidance [16].

Some researchers also fabricated surgical stents using stereolithographic (.stl) models [17,18]. When computed tomography (CT), cone-beam Computed Tomography (CBCT), Computer-Aided Design (CAD), Computer-Aided Manufacturing (CAM) and 3D printing technology became available and easy to use, they were considered tools for exact placement of mini-implants. CBCT imaging for exact mini-implant guidance and implementation has been used by several researchers [19,20,21,22,23]. In order to combine information about the condition of the soft and hard tissue, others have used CBCT in addition to intraoral scans and scans of dental casts. Surgical guides have been designed on digital models created by merging Digital Imaging and Communications in Medicine (DICOM) and .stl files [24,25,26,27].

As a result of these studies, 3D technology in imaging, designing and manufacturing, have assisted in accurate placement of mini-implants. However, some of the studies mentioned above utilized the CBCT only for determining the exact point of insertion and not for designing a surgical guide [23]. Others used procedures which may compromise accuracy, such as measurements on DICOM files transferred to the surgical site [22] or incorporating metal sleeves or pathways to the stent for guiding the driver and not for guiding the mini-implant itself [26,27]. Therefore, there is a need for simplifying the procedures of 3D designing of a surgical guide, which will serve every mini-implant type and lead to easy adaptation on the patient’s mouth for exact implementation.

The purpose of this study was to evaluate the accuracy of mini-implant placement according to a pre-planned position (through linear and angular measurements) with the assistance of a computer designed and 3D printed surgical guide. The hypothesis is that such a surgical guide with the use of CBCT would lead to a more accurate placement of a mini-implant than a surgical guide fabricated without the use of a CBCT by only evaluating clinical and 2D radiographic information.

## 2. Materials and Methods

In this study 35 mini-implants (Aarhus, Medicon eG: *n* = 20, Ø: 1.5 mm and Abso Anchor, Dentos Inc.; *n* = 15, Ø: 1.3–1.4 mm), were placed in 15 patients aged between 13 and 26 years old, who were under orthodontic treatment in the orthodontic clinic of 401 General Army Hospital in Athens, Greece and in private orthodontic office in Athens, Greece. The inclusion criteria were: (a) patients with a CBCT scan (which was acquired because impacted teeth were involved or moving certain teeth according to treatment plan introduced questioning on safety) and (b) the need to use mini-implants for anchorage purposes. Exclusion criteria included poor oral hygiene, periodontal disease, metabolic disease affecting healing (diabetes), and smoking.

In 11 patients, two mini-implants were placed (right and left side of the same jaw), in three patients four mini-implants (two in the upper and two in the lower jaw, right and left) were placed, and in one patient only one mini-implant was placed. During treatment, one mini-implant became loose and was lost, so it was excluded from the study. For 18 of the 34 mini-implants (study group), CBCT imaging and intraoral scanning were used for deciding the exact position and inclination and for designing a surgical guide. For the other 16 (control group), information on panoramic imaging (without the use of the CBCT) and clinical evaluation in combination with intraoral scanning was used. In this case, mesial and distal contour of adjacent teeth were used for determining the septum midline as a reference for mini-implant insertion, as suggested by Estelita et al. [28]. Inclination was set according to clinician’s perception as to occlusal plane. The need for a surgical guide even in this later approach (instead of immediate insertion) was required, as inaccuracy can occur due to mini-implant’s tip slip or soft tissue pressure on the screwdriver, during operation. The patients or their parents were fully informed about the exact procedure, and the aim of the study, and signed a consent form. In the form, information was provided concerning the need of mini-implants for the case and the reasons for acquiring a CBCT image. Voluntary participation was highlighted and so was the option to withdraw from the study at any time without having a negative impact on the treatment provided. The study protocol was approved by Scientific and Ethics Committee of 401 General Army Hospital in Athens, Greece (ref: No 10/8-12-2020).

### 2.1. Design of the Surgical Guide

**Figure 1:**
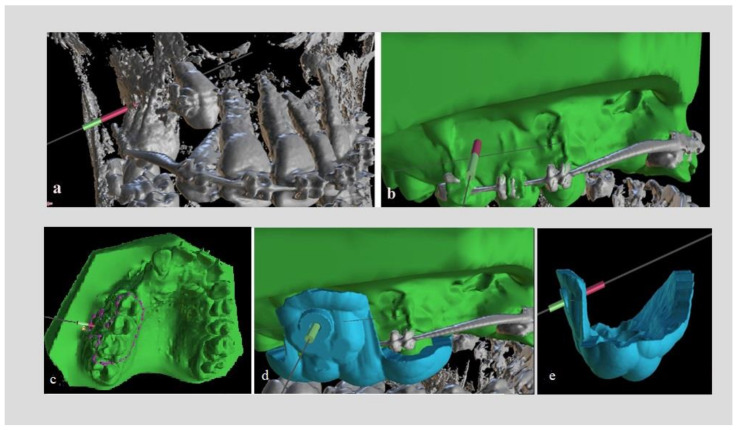
(**a**) Cone-Beam Computed Tomography (CBCT) image with virtual mini-implant in place. (**b**) Digital model A: Digital Imaging and Communications in Medicine (DICOM) and stereolithography (.stl) files combined. (**c**) Design of the surgical guide on the digital model. (**d**,**e**) .stl model of the surgical guide.

Several months after initiation of an orthodontic treatment and when stainless steel, rigid rectangular orthodontic wires were in place, the procedure for placing mini-implants started. Patients were first subjected to CBCT imaging of the jaw that mini-implants were scheduled to be placed (Planmeca ProMax^®^ CBCT system, 90 kVp/4–10 mA, 200–400 μm voxel size). Digital scanning of the dental arch was also performed with a Carestream 3600 digital oral scanner, incorporating soft tissues, and a .stl file was acquired. For the 18 mini-implants, which served as study group (alternately selected either for the right or left side of the patient, one after the other), DICOM and .stl files were uploaded on open-source software (Blue Sky Plan^®^). This software is utilized for viewing and reformatting images created by computerized tomography and can be used for virtual implant implementation and surgical guide fabrication. The two files were combined with several matching points (mesial and distal points on incisal edges of incisors) on to a digital model (Digital Model A) in which model roots, bone, mucosa, and teeth could be evaluated at the same time. Then, virtual mini-implants from the program panel were inserted at the desired position. These were custom designed with the diameter and length of the real mini-implants that were scheduled to be implemented (Figure 1a,b). The area of the mini-implants placement (mostly buccally between first and second molar or second premolar), the inclination to the occlusal plane and the point of insertion were selected upon treatment goals.

For the 16 mini-implants in which CBCT was not utilized (control group), the procedures were exactly the same as in producing the combined presurgical digital model. The difference was that the surgical guide was designed on the .stl model by evaluating the panoramic x-ray and clinical information transferred to that model. Specifically, this clinical information comprised stamps made on gingival tissue by dental floss pulled apically, following the surface contour of the teeth. These stamps were recorded during intraoral scanning, and, in this way, a clinical reference representative of the septum limit was created. In this way, the septum midline was determined for safe implementation. CBCT information was not used in this case.

The design of the surgical guide was done by drawing a peripheral line on the digital model, mostly leaning on occlusal surfaces with a projection to the area of placement (Figure 1c). The thickness of the guide was set to 2 mm. Guiding holes for mini-implants were incorporated on the guide and the diameter was set exactly as the diameter of the real mini-implant (1.5 mm-1.4 mm-1.3 mm). The length of the guiding holes was designed to be 3 mm, providing satisfying guidance for placing the mini-implant (Figure 1d). The surgical guide was then exported as a .stl model (Figure 1e) and then 3D-printed in a biocompatible resin on a Formlabs Form 2 3D-printer.

### 2.2. Surgical Procedure

**Figure 2:**
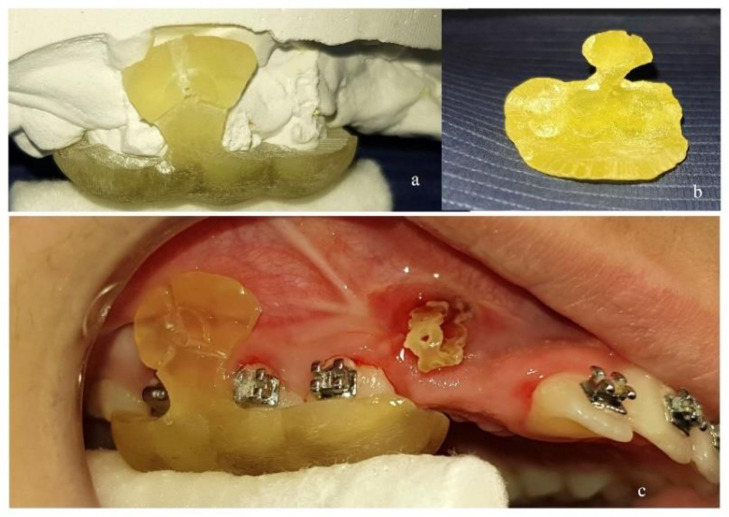
(**a**) Surgical guide 3D-printed in biocompatible resin and tested for proper fitting on plaster model. (**b**) Inner side of the surgical guide. (**c**) Surgical guide tested for proper fitting in patient’s mouth.

The surgical guide was first tested for proper fitting on a plaster model of the patient’s dental arch (Figure 2a,b) and then on the teeth and mucosa in the patient’s mouth (Figure 2c). Care was taken so that the edges of the surgical guide would not interfere with molar tubes or brackets. For that purpose, proper grinding was performed. This is of great importance, as design of the surgical guide on the digital model imposes some compromises concerning contact of the guide with fixed appliances.

**Figure 3:**
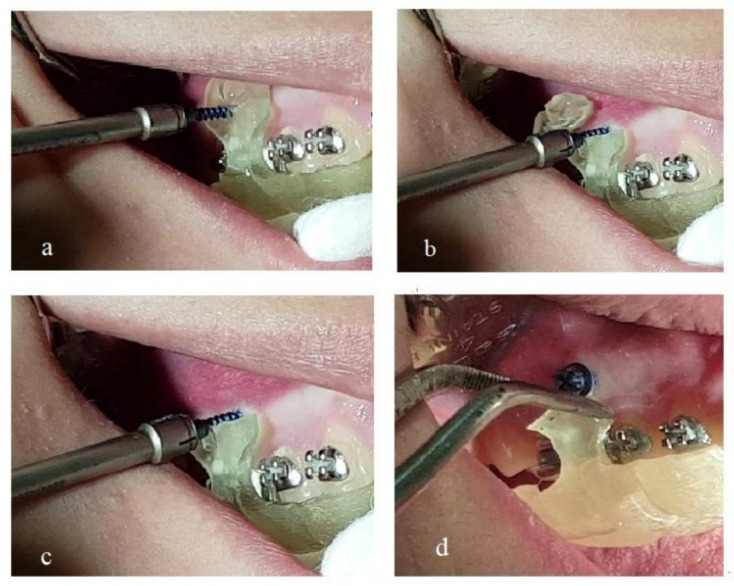
(**a**) Mini-implant inserted through the guiding hole of the surgical guide. (**b**) Guide’s small part beyond the mini-implant broken to pieces under screwing pressure. (**c**) Broken pieces of the guide removed. (**d**) The guide released from the mouth.

After local anesthesia, the mini-implant was inserted through the guiding hole and driven to the designed position and inclination (Figure 3a). By continued screwing, the mini-implant perforated the mucosa and finally the bone. The procedure continued until the mini-implant’s neck had reached the outer limit of the guiding hole. Three radially incorporated grooves to the guide were designed, starting from the guiding hole and reaching the upper (or lower), mesial and distal edge of the guide (Figure 2a,c). By continued screwing from that point, the guide’s small part beyond the mini-implant was broken into pieces under pressure (Figure 3b) and was removed (Figure 3c). In this way the guide could be easily pulled down by dental forceps and released from the mouth, as there was no other interference (Figure 3d).

**Figure 4:**
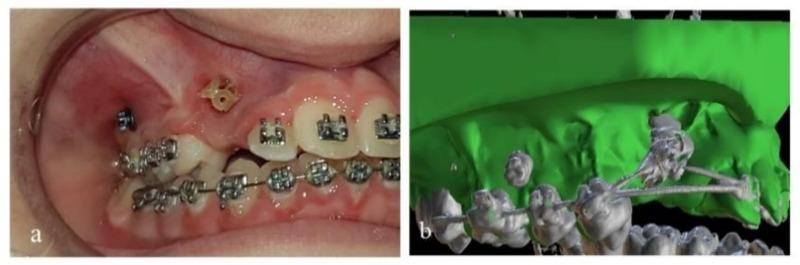
(**a**) Mini-implant in final place. (**b**) Digital model B (Ιnitial .stl model and post-surgical DICOM files combined).

After that, the mini-implant was inserted to its proper final position (Figure 4a).

After several (usually 4 to 6) months, and when desired orthodontic movement with mini-implants’ assistance was completed, new CBCT imaging was acquired while the mini-implants were still in place. The purpose was to check if impacted teeth or certain teeth had moved away from adjacent roots with safety. Precaution was taken so that no change in the orthodontic archwire occurred, and that the central incisors were not moved between the two CBCT radiographs, as they served as the reference for combining the new DICOM images with the original .stl model (Blue Sky Plan 4, Software), using the same procedure and matching points on the incisal edges. As a result, a new digital model (B) was formatted (Figure 4b) and the same measurements were acquired so as to evaluate the mini-implant’s position by comparing the digital planned position and the actual position of mini-implants in patient’s mouth.

### 2.3. Measurements for Mini-Implant Position

In digital model A, having on-site CBCT images and just the contour of the .stl model, a coronal slice on the point of insertion of the virtual mini-implant in the bone and an axial slice in which most of the mini-implant’s body was visible, were evaluated.

**Figure 5:**
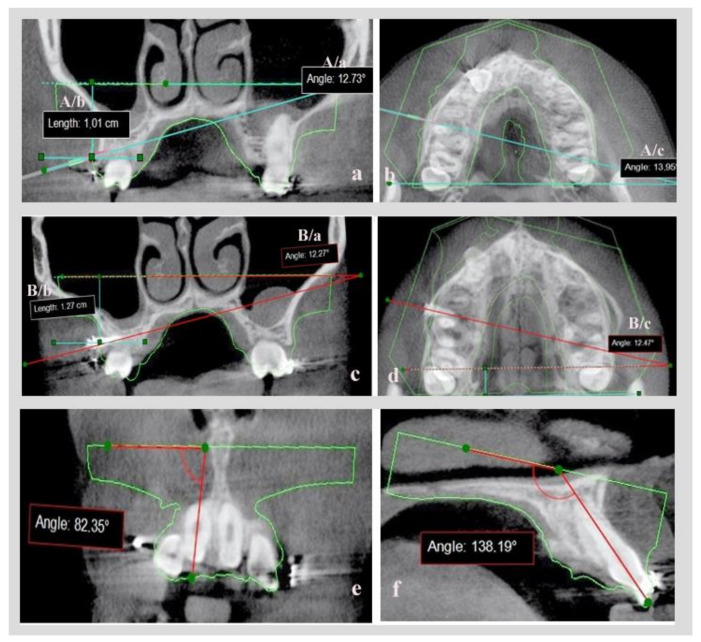
(**a**) Coronal slice (measurement set A/a, A/b). (**b**) Axial slice (measurement set A/c) for evaluating virtual mini-implant’s position. (**c**,**d**) Coronal and axial slice for evaluating the real mini-implant’s position (measurement set B/a, B/b, B/c). (**e**) Angle of axial line of central upper right incisor and the base of the STL model in coronal slice. (**f**) Angle of the axial line of the central upper right incisor and the base of the STL model in a sagittal slice.

On the coronal slice, measurement was made of the angle defined by the axial line of the virtual mini-implant and the lower base of the .stl model and of the distance of the point of insertion in the bone, to the lower base of the .stl model (Figure 5a: measurement set A/a = coronal angle, A/b = coronal distance). An axial slice measurement was made of the angle defined by the axial line of virtual mini-implant and the lower base side of the .stl model (Figure 5b: measurement set A/c = axial angle).

The same set of measurements were calculated on digital model B with real mini- implants already inserted (Figure 5c: measurement set B/a = coronal angle, B/b = coronal distance, Figure 5d: measurement set B/c = axial angle). By comparing the A and B sets of measurements, and after proper statistical analysis, the accuracy of the mini-implants’ placement could be evaluated after utilization of CBCT or not.

In order to validate the reproducibility and the accuracy of combining the initial .stl and DICOM files before and after mini-implant placement, while creating digital models A and B, two angles were utilized: the angles formatted by the axial line of the central upper or lower right incisor and the base of the initial .stl model in a coronal (Figure 5e) and a sagittal slice (Figure 5f).These were calculated on models A and B and compared, since movement of central incisors was avoided between the initial and the final CBCT.

### 2.4. Statistical Analysis

SPSS software was used for all statistical analyses (Statistical Package for the Social Sciences, SPSS 25.0, Inc.-Chicago, IL, USA). The Shapiro-Wilk normality test was used to determine normal distribution. T-tests were used in the case of normal distribution and the Mann-Whitney test was used in the case of not normal distribution. The results were significant at *p* < 0.05.

All measurements were performed by two investigators. Inter-rater reliability was assessed by ICC test.

## 3. Results

The ICC test showed excellent inter-rater reliability (0.988).

For the patients in which CBCT was utilized for mini-implant placement (study group) there was no statistically significant difference in any (angular or linear) of the measurements between the two different time points; pre-op (before the real mini-implants’ placement, after the 3D placement of the virtual mini-implant) and post-op (after the completion of the orthodontic movements with the real mini-implants inserted in the patients’ mouth). Specifically, the *p*-value was found to be 0.061 for the coronal angle, 0.412 for coronal distance and 0.212 for axial angle (Table 1).

For the patients in which CBCT was not used for mini-implant placement (control group), there were statistically significant differences for the two angles that were measured. More specifically, the coronal angle was set to 13.6° pre-op and was measured 22.7° post-op (*p*-value: 0.002) and the axial angle was set to 13.4° and was measured 15.9° (*p*-value: 0.034). Linear measurements had no statistically significant differences pre (1.1 cm) and post-op (1.3 cm)-Table 1.

The differences in each measurement pre-op and post-op were calculated in both groups (Table 2), and a statistically significant difference was found for the coronal (*p*-value: 0.025) and for the axial angle (*p*-value: 0.020).

Finally, no statistically significant differences were found between measurements that were used to validate the accuracy of combining the initial .stl file and DICOM files pre- and post-op, in this study (Table 3).

## 4. Discussion

Results of this study demonstrate that CBCT imaging and intraoral scanning can be used in combination for designing a surgical guide for accurate mini-implant placement, and support the hypothesis stated. Specifically, we found that the point of insertion of mini-implants in the mucosa (defined as linear measurement to the base of the initial .stl model) was accurately determined in both the study and control groups. However, the direction of the mini-implant (defined by angular measurements in coronal and axial slices as to the base of the .stl model) was well determined only in study group, in which the CBCT scan was taken into consideration in conjunction with the intraoral scan. In the control group, statistically significant differences were found in both angles measured, but the difference of 2.5°, which was found for the mean value of the axial angle, cannot be considered clinically significant. Procedures in this study were simple in contrast to others [25,26,27].

When surgical guides are fabricated with acrylic resin on dental casts [12], or when simple wire stents are used [15], only the point of insertion is determined and not the exact pathway of the mini-implant through the bone.

In the study of Kalra et al. [22], the distance between the distal end of the second premolar bracket and the point of intersection of the perpendicular from point of insertion to the archwire was measured using CBCT imaging in order to transfer the ideal site clinically. Likely in the study of Landin et al. [23], CBCT was used for determining the insertion point, which was found at the mid root area and then at the center of the buccolingual alveolar ridge, after evaluating cross-section images. These procedures, however, provide information only for the point of insertion and not for the inclination of the mini-implant.

Bae et al. [25] investigated the accuracy of mini-implant placement in cadaver maxillae using computer-aided surgical guides. Impressions and CBCT images of the cadaver maxillae were undertaken. The digital models, created by scanning of the plaster models, and the CBCT images were fused to create a 3D digital image. Mini-screw placement planning was conducted in the 3D images and then used for computer-aided surgical guide fabrication. Finally, the surgical guides incorporated metal sleeves for driver adaptation, predrilling and finally drilling. In a quite similar procedure, Wang et al. [26] utilized CBCT, digital laser scanning from stone models, computer-aided (CAD) system and 3D printing to fabricate an accurate surgical guide for mini-implant placement. Guide cylinders were designed in the guide for driver adaptation. However dental cast fabrication and then scanning imports a certain deviation in merging CBCT and scanning images [29]. To avoid such deviations intraoral scanning was advocated in this study, so that soft tissue registration and teeth matching on the DICOM and .stl files were more accurate. Another difference is that while in the above-mentioned studies guiding metal sleeves and cylinders for driver adaptation were used, in our study a certain tunnel, with a diameter identical to the mini-implant, was computer-designed and used for implant adaptation and guiding.

Ιn the study of Yu et al. [27] CBCT was used for the design of a surgical guide without the use of a dental scan. For soft tissue evaluation, the authors used aluminum wires attached in a vacuum splint from dental casts. Similar to our study, a specific guide for implant placement was designed in addition to guided cylinders for the driver. The difference, though, is that in our study soft tissues were captured with the intraoral scan and we incorporated strategically positioned grooves in our guide design, permitting a small piece of the guide beyond mini-implant to break under driving pressure allowing the easy removal of the guide, while Yu et al. [27] used spin off slots for guide removal.

Additionally, in our study, the initial .stl model was used as a connecting element between pre-op and post-op CBCT. It was also used as a reference tool for angular and linear measurements in recording mini-implant position. This was feasible because the central incisors were left immovable on purpose (no movement and no archwire change) between the two CBCT radiographs, and certain points on their incisal edges were used for merging DICOM and .stl files. To evaluate the method of combining .stl and DICOM files used in this study and to ensure that digital models A and B were comparable, the angles formatted by the axial line of central upper or lower right incisor and the base of the .stl model in a coronal and in a sagittal slice were utilized and compared, (Figure 5e,f) and no difference was found between models A and B (Table 3).

The accuracy of mini-implant placement postoperatively has been evaluated in some studies by radiographs [16,26] and in others with a postoperative CBCT [19,20,21,23,24,25]. Radiographs confirmed the safe placement of mini-implants, but they did not verify the inclination or the exact position of mini-implants. A postoperative CBCT seems to be more appropriate for that, and concerns for possible excessive radiation dose should be considered in regard to a safe and effective implementation, especially when alveolar boundary conditions or treatment outcome in regard to safety of adjacent roots need to be considered [30]. This is the reason for selecting limited field of view (FOV-Ø 5 × 8 cm or Ø 8 × 8 cm) for CBCT imaging in this study. A low dose protocol mode, with a low radiation dose, pre and post -op, was also utilized.

Finally, in this study the second CBCT was taken, after desired movement of teeth with mini-implant assistance was achieved, for evaluating safety (as impacted teeth were moving away of adjacent roots) and not just after implantation for evaluating the accuracy of mini-implant placement. However, to do so, and for digital models A and B to be comparable, any active movement for central incisors and any archwire change between pre- and post-op CBCT were avoided. In this way we were able to combine the initial .stl file with pre- and post-op DICOM files, and the digital models A and B that were produced were comparable for evaluating mini-implant position. This was proven accurate by comparing the angles formatted by the axial line of central upper or lower right incisor and the base of the initial .stl model, in a coronal and a sagittal slice, between digital models A and B. No statistically significant differences were found for those angles, which proves the accuracy of the method.

In summary, the surgical guide used in this study, which was designed and manufactured using technologies such as intraoral scanning, CBCT imaging, special computer software and 3Dprinting, can overcome inaccuracies introduced by designing the guide on plaster models or incorporating elements which compromise point of insertion, exact pathway and inclination of the mini-implant. The method advocated is simple and easy to perform. As to study design, care must be taken on merging DICOM and .stl files. This can be automatically performed by the software used, but manual adjustments may sometimes be required. This is of great importance, as angular and linear measurements pre- and post-op, which are used for determining accuracy in mini-implant placement, may not be comparable.

## 5. Conclusions

The need for accuracy in mini-implant placement can well be served by a surgical guide designed with the help of a CBCT of the patient’s jaw and intraoral scanning of corresponding dental arch. The combined DICOM and .stl files can be used to produce a 3D digital model in which the point of insertion of the mini-implant, inclination and distance to adjacent anatomic structures can be determined. On the other hand, a 3D printed surgical guide designed without CBCT acquired information, but with clinical evaluation transferred on a .stl model of the dental arch, accompanied by 2D radiographic information, is accurate concerning point of insertion but not with respect to desired inclination of the mini-implant. Computer-aided design of the guide and 3D printing facilitates fabrication of the guide, while incorporating special guiding holes and grooves on the guide lead to accurate positioning of the mini-implant and convenient release of the guide. The use of a certain limited FOV of the upper or lower jaw, and not full head CBCT, along with a low dose mode, can effectively reduce the radiation absorbed by the patient.

## Figures and Tables

**Table 1:** Measurements for the study group (*n* = 18) and control group (*n* = 16) pre- and post-op.

Groups		Pre-op				Post-op				*p*-Value
		Min	Max	Mean	SD	Min	Max	Mean	SD	
**Study group**	**Coronal angle (°)**	5.1	25.8	**14.7**	6.0	7.6	32.9	** 17.1 **	5.8	0.061
	**Coronal distance (cm)**	0.7	2.4	** 1.2 **	0.4	0.7	2.7	** 1.3 **	0.5	0.412
	**Axial angle (°)**	4.2	28.2	** 14.6 **	6.5	2.1	27.5	** 13.3 **	8.3	0.212
**Control group**	**Coronal angle (°)**	1.0	23.6	** 13.6 **	5.7	5.2	44.6	** 22.7 **	11.2	**0.002**
	**Coronal distance (cm)**	0.7	1.7	** 1.1 **	0.3	0.8	2.4	** 1.3 **	0.4	0.400
	**Axial angle (°)**	7.6	22.1	** 13.4 **	4.1	3.1	41.0	** 15.9 **	10.3	**0.034**

**Table 2:** The differences between the measurements pre-op and post-op in both groups.

	Study Group				Control Group				*p*-Value
	Min	Max	Mean	SD	Min	Max	Mean	SD	
**Coronal angle (°)**	0.5	10.6	** 4.6 **	3.0	0.4	33.8	** 10.4 **	8.5	**0.025**
**Coronal distance (cm)**	0.01	1.2	** 0.2 **	0.3	0.02	1.1	** 0.2 **	0.3	0.384
**Axial angle (°)**	0.01	15.3	** 4.6 **	4.9	0.48	26.3	** 9.4 **	6.4	**0.020**

**Table 3:** Measurements to evaluate accuracy in combining .stl and DICOM files.

Incisors	Angle	Pre-op				Post-op			
		Min	Max	Mean	SD	Min	Max	Mean	SD
**Maxillary (*n* = 13)**	Coronal (°)	78.1	90.9	**84.3**	4.3	78.9	90.9	**85.0**	3.8
	Sagittal (°)	17.8	138.2	**125.6**	7.0	118.2	137.9	**125.8**	6.1
**Mandibular (*n* = 5)**	Coronal (°)	85.0	100.0	**89.6**	6.1	85.8	98.9	**89.5**	5.4
	Sagittal (°)	93.0	131.2	**113.7**	15.3	91.9	128.3	**112.4**	14.3

## Data Availability

Data available upon reasonable request.
